# Supplementary material for: Valuation of Ecosystem Services for the Sustainable Development of Hani Terraces: A Rice–Fish–Duck Integrated Farming Model
Source: Int J Environ Res Public Health. 2022 Jul 13;19(14):8549. doi: 10.3390/ijerph19148549 (PMC9317269; doi:10.3390/ijerph19148549)
Supplement: Supplementary file 1 [file ijerph-19-08549-s001.zip › ijerph-1741211-supplementary.pdf]

Questions for farmer

Name of village: Phone number:

1. Name, gender, age, education, number of family.
2. Number and size of land owned. How many are in actual operation?
3. Integrated rice-fish-duck farming areas? Hybrid rice culture areas? Red rice culture areas? Fish farming areas?
4. Who is doing day-to-day operations on the land? How many years are engaged in the farming?
5. What are the main activities on the farm?
6. Some monitoring data

The height of the ridge	
daily water depth in the terraces	
number of the growth periods of rice	
number of the growth periods fish	
number of the growth periods duck	
the number of days of standing water during the growth period of rice	
the number of hot days in summer	
market price of agricultural water	

7. The input and harvest of rice-fish-duck farming in 2020

		Volume/Yield	Price
**Input**	Land rent		
	Seed of rice		
	Seed of fish fry		
	Seed of ducking		
	Fertilizer		
	Pesticide		
	Labor		
**Harvest**	Rice		
	fish		
	duck and duck egg		

8. Is additional labour hired in peak season or for harvest? If yes, what are the costs?
9. Where and how was the product sold? Bought by whom? (trader, individual consumer, processing company etc.)

10. What are main problems / challenges experienced in farm? (techniques, accessibility of inputs, markets and price for harvested products, support, credit, feed, seed, etc.).
11. Integrated rice-fish-duck farming has various ecosystem services such as climate regulation, water conservation, and biodiversity maintenance. Do you know this?
12. In your opinion, what other aspects of the integrated rice-fish-duck farming ecosystem of Hani terraces need to be improved?
13. Any other comments you would like to give? (any other remark in relation to the project).

Thank you for your time!
